# Safety Assessment Review of a Dressing Assistance Robot

**DOI:** 10.3389/frobt.2021.667316

**Published:** 2021-06-14

**Authors:** Daniel Delgado Bellamy, Gregory Chance, Praminda Caleb-Solly, Sanja Dogramadzi

**Affiliations:** University of the West of England, Bristol, United Kingdom

**Keywords:** assistive robotics, collision avoidance, SHARD, system-theoretic process analysis, unified modeling language, pHRI, safety, industry standards

## Abstract

Hazard analysis methods such as HAZOP and STPA have proven to be effective methods for assurance of system safety for years. However, the dimensionality and human factors uncertainty of many assistive robotic applications challenges the capability of these methods to provide comprehensive coverage of safety issues from interdisciplinary perspectives in a timely and cost-effective manner. Physically assistive tasks in which a range of dynamic contexts require continuous human–robot physical interaction such as e.g., robot-assisted dressing or sit-to-stand pose a new paradigm for safe design and safety analysis methodology. For these types of tasks, considerations have to be made for a range of dynamic contexts where the robot-assistance requires close and continuous physical contact with users. Current regulations mainly cover industrial collaborative robotics regarding physical human–robot interaction (pHRI) but largely neglects direct and continuous physical human contact. In this paper, we explore limitations of commonly used safety analysis techniques when applied to robot-assisted dressing scenarios. We provide a detailed analysis of the system requirements from the user perspective and consider user-bounded hazards that can compromise safety of this complex pHRI.

## I Introduction

Assistive robotics is increasingly becoming recognized as a potential enabler in helping people improve their quality of life and live independently, particularly in later life (Zafrani and Nimrod, 2019). Fundamental to the successful design and deployment of any assistive solution for non-expert users of robotics is ensuring safety. Therefore, the focus of this paper is on methods for hazard analysis used for safety assurance during close proximate physical Human–Robot Interaction (HRI). We have considered user requirements and scenarios in the context of robot-assisted dressing.

We know that in other application domains such as the use of industrial co-bots for manufacturing (El Zaatari et al., 2019), safe HRI has been possible due to existing regulations that dictate limited contact and compliant physical interaction (Haddadin et al., 2008). However, from a practical point of view, industrial robotic standards, lacking consideration of direct physical contact between the person and the robot, are not suitable for physically assistive robots. Another added challenge is that industrial regulations normally count on trained users that could supervise the robot’s operation with expert intuition and understanding of safety guidelines. In domestic environments, assistive technologies are likely to be used by people (for example, frail older people, physiotherapists, carers) who are currently not expected to have the similar experience and training as engineering experts. As such, where domestic applications are concerned, safety standards and safety assessment approaches need to be reconsidered for their appropriateness as we adapt robotics to new applications within health and social care, accounting for extended physical Human–Robot contact and non-expert service users.

**FIGURE 1:**
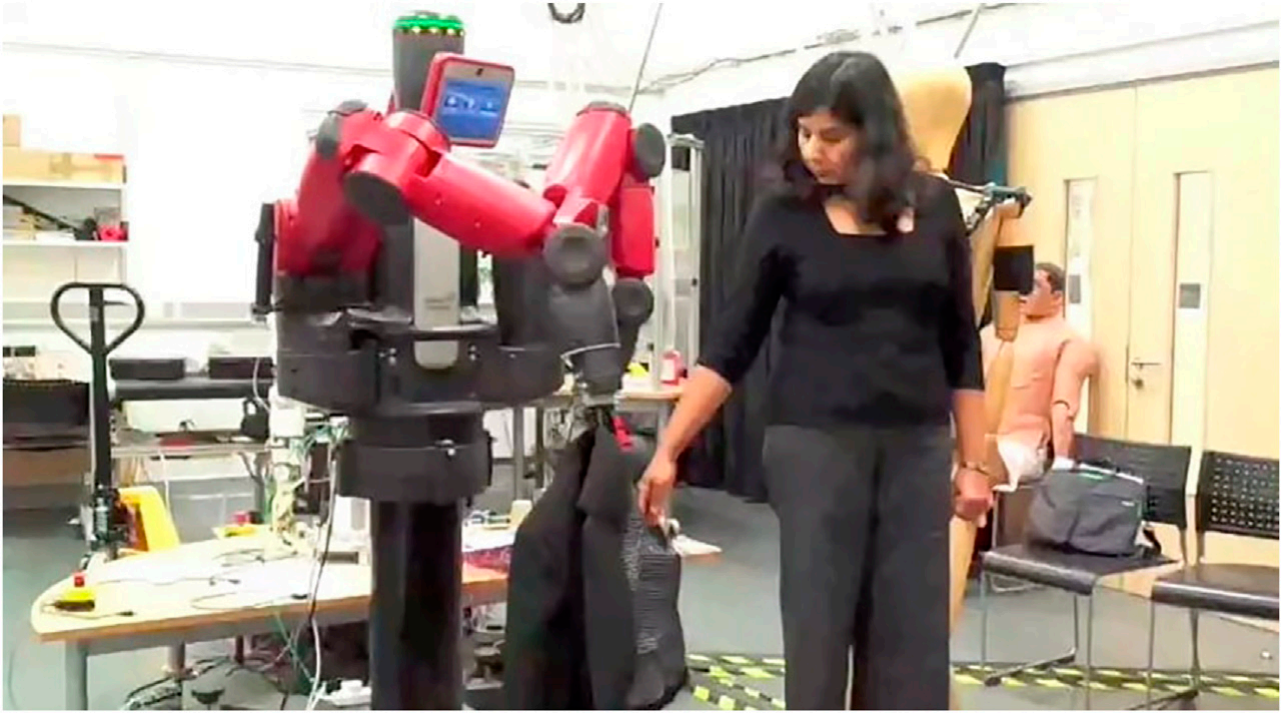
Dressing assistance task performed by a robot.

This paper is structured into 5 sections. In Section I we introduce the motivations for this paper. Section II reviews existing hazard analysis methods and critically overviews safety of existing physical Human–Robot Interaction (pHRI) studies, emerging standards for pHRI design and user modeling in this context. In Section III we present a pHRI for dressing assistance use case (see Figure 1)and apply two hazard assessment approaches (SHARD-UML and STPA) to analyze safety. Lastly, in Section IV, we explore safety measures to mitigate the limitations identified, drawing on human factors, physical Human–Robot Interaction modeling for control and monitoring, emerging approaches for collaboration between the user and robotic systems and inter-disciplinary design approaches. Our aim is to identify and raise awareness of the safety challenges that need to be considered during pHRI in assistive robotics applications, so that they can be addressed and resolved earlier in the development stage.

The specific contributions of this paper to the research community include: a review of the existing hazard analysis methods and ISO regulatory guidance; an investigation that queries if these tools are fit to purpose for our use case when PHRI is a requirement; a method for analyzing the outcomes of hazard techniques and evaluate their scope and limitations; and guidance for similar pHRI use cases that struggle to implement hazard mitigation and safety guidance, and still meet their application pHRI requirements.

## II Literature Review

This section reviews some of the conventional hazard assessment approaches and techniques applied to pHRI safety analysis, and discusses their shortcomings, particularly in relation to the assisted dressing use case being considered in this paper.

### A Hazard Assessment for pHRI

Well-established tools for assessing safety in industrial processes date back to the 1950’s, and they are still included in the current guidelines, for example, ISO31010 (IEC 31010, 2019). The most notable techniques are FMEA (Failure Mode and Effects Analysis) (IEC 60812, 2018); HAZOP (HAZard and Operability study) (BS EN 61882, 2019) and its variant SHARD (Software Hazard Analysis and Resolution in Design) (Pumfrey, 1999), and STPA (Systems Theoretic Process Analysis) (Leveson, 2004). The bottom-up nature of these methods provides comprehensive coverage of failures by scrutinizing every component of the system in industrial applications typically using extensive expert knowledge to review the system at a fine level of granularity. With increasing complexity of robotics systems, this approach may become unfeasible. To embed safety and trust in pHRI, knowledge from different disciplines is now required (Salem et al., 2015; Eder et al., 2014). Moreover, hazard analyses normally take place before or in the proof of concept phase to facilitate efficient design early in the conception of the system. Even at this stage there may be incomplete information to assess issues emerging from deployment in real-world environments (Bolbot et al., 2019). Both HAZOP (BS EN 61882, 2019) and FMEA have also been found to be limited when it comes to their application in complex systems which are highly inter-linked (Bolbot et al., 2019). There are other hazard analysis techniques such as STPA that claim to provide a wider coverage of the development process and address not only physical failures, but also potentially unsafe behaviors (Leveson, 2004). An STPA analysis for the “Lane change” action of an Automated Driving System [Cruising Chauffeur^®^ with Safety in Use (SiU)] (Abdulkhaleq et al., 2018) acknowledged some improvement during the brainstorming process and a multi-interaction consideration in early stages of development (driving system, user, and external agents). Other research studies have preferred STPA over other traditional hazard assessment methods to evaluate multi-robot mobile system risk level (Bensaci et al., 2018). However, STPA still requires engineer’s expertize to be adapted and completed, and it can be more exhaustive than traditional methods due to the higher level of variables to assess (Bolbot et al., 2019).

A number of researchers have combined traditional hazard analysis techniques such as the HAZOP+UML approach (Do Hoang et al., 2012; Guiochet, 2016) to make them more relevant for pHRI analysis. By combining these techniques they found that they were able to get more comprehensive and rigorous coverage of risks. In the European Commission funded project SAPHARI (2011–2015) (De Luca, 2015), researchers successfully used more recent versions of HAZOP and UML techniques (Guiochet et al., 2012) in pHRI studies that feature collision avoidance in industrial settings (Guiochet, 2016). A notable attempt to classify pHRI according to the interaction contact type is considered in (Guiochet et al., 2017) defining continuous pHRI as ’physical interaction that occurs continuously over extended periods of time’. This term was defined for industrial robots which does not necessarily address complexity of physical assistive robots.

In physical care assistance robotics, the presence of vulnerable users with different health conditions adds an additional layer of complexity. This is due to a high level of variance emerging from the range of health conditions which need to be represented in user modeling. If dynamic user profiles and real-world environments are part of the system under risk consideration, HAZOP and FMEA are not suitable for hazard assessment as they would miss errors produced by the synthesis of models from different disciplines such as engineering and healthcare (Salem et al., 2015).

Additionally, the impact that these conditions have on the users’ physical and cognitive abilities are also likely to affect the types of risks that need to be considered. As robots migrate from the shop floor in industrial applications to care homes in an assisted living setting, so must the regulations that govern them to comply with more niche user requirements such as psychological support among other things (García-Soler et al., 2018).

In industry, the risk assessment also accounts for human factors by using Human Reliability Analysis where the behavior model of operators, engineers, users, and others human agents is considered to mitigate hazards. Methods such as HAZOP and STPA that focus on the system structure also infer similar hazards but they address human errors indirectly. If many accidents have been caused by long-term organisational or managerial problems that are lacking human factor attention (Fossum et al., 2018), traditional techniques such as HAZOP and STPA also lose coverage for this reason.

We observed how some studies propose the insertion of HRA (Hazard Risk Analysis) in healthcare with influential factors including Environment, Software, Hardware, and Liveware (Onofrio and Trucco, 2018) (where Liveware includes categories such as fatigue, familiarity, workload, and communication, among others). Regarding robotic healthcare applications, expert operators may not be constantly present during the service execution for real world environments, delegating the handling and responsibility of potentially unsafe situations to the user.

### B Emerging Standards and Regulations for the Design of pHRI

Robot design is currently regulated by the ”ISO/TC 299—Robotics” previously known as ”ISO/TC 184—Industrial automation” when conceived in 1983. Physical contact safety is addressed for industrial collaborative robots, AKA co-bots using the ISO 15066:2016 (Haddadin et al., 2008); and for Personal care robots using the ISO 13482:2014 (ISO 13482, 2014) (including only exoskeletons as physically assistive robots). The consolidation of pHRI safety regulations for these standards have been mainly supported by the PHRIENDS EU project (2006–2009) and, by its successor: SAPHARI project (2011–2015).

The concern is that reference standards still assume deterministic and known actions and may lose effectiveness as the application starts to involve more non-deterministic or unpredictable actions. For instance, emergency stops or reactive approaches are frequently recommended by regulations to avoid collision hazards whereas, in healthcare scenarios, they could result in harming the user or other objects, for example if the robot is in the process of dressing a person or helping them to keep their balance. While there are many studies which have addressed collision avoidance as per the regulations, such as in the PHRIENDS project (http://www.diag.uniroma1.it//∼labrob/research/PHRIENDS.html#Papers) (Haddadin et al., 2008; Haddadin et al., 2009; Albu-Schäeffer et al., 2008; De Luca et al., 2006; De Luca et al., 2009), and SAPHARI project (http://www.saphari.eu/index.php?option=com_content&view=article&id=120:publications-2&catid=29&Itemid=185) (De Luca and Flacco, 2012; Beetz et al., 2015; Zube, 2015; Walther and Guhl, 2014) through reactive control and compliance design toward lightweight robots with variable stiffness, their focus remains on human–robot collision avoidance. These studies mostly consider physical human robot interaction within the industrial environment, generally considering the presence of physical contact as a hazard to the user’s safety. However in certain physical assistance tasks, physical contact is a key feature of the interaction, but we have not found any safety standards or regulations which provide guidance relating to this.

### C Current Approaches for Exploiting Physical Human–Robot Contact to Improve Interaction

In most industrial contexts, physical human–robot contact is avoided and contact between humans and robots is considered a safety hazard. There is scope however, to consider pHRI as a potential means of communication. The SAPHARI project introduced a prototype of a tactile sensor multi-array that perceives the intensity of the force and its location with a certain resolution (Cirillo et al., 2016).

This led us to investigate what has been done to implement continuous physical contact interfaces in the literature, particularly those inspired by Human-Human Interaction (HHI). A number of studies have explored the role of touch-force in cooperative human–robot tasks to extract features that could be useful in HRI as a meaningful source of information. Takagi et al. (2018) considered how the user’s comfort can be maximized by modeling force feedback from the user, Nishimura et al. (2015) investigated how the cooperation status level for assistance can be optimized based on user force feedback too, Mortl et al. (2012) and Gienger et al. (2018) explored how force information can be used for active and passive role allocation changes in cooperative tasks, and Ansari et al. (Ansari et al., 2018) considered how the user’s hand orientation can be estimated when carrying an object with the robot. Although these studies still need more empirical evidence, they have provided new insights on how physical contact can be exploited to support pHRI, identifying how soft and hard interactions occur at different points of a physical interaction and how a physically assistive robot could make use of this information.

A further example of using pHRI for communication was presented in a robotic wheelchair study where haptic force, combined with artificial intelligence learned haptic control policies from Human–Human Interaction demonstrations (Kucukyilmaz and Demiris, 2018). Even though no significant difference was noticed in terms of effort and performance when haptic feedback was applied, higher levels of comfort and enjoyment among the participants in the interaction were identified, which accounted for long-term psychological effects. In another assistive application example, force feedback and vision were used to improve the estimation of the user’s trajectory, which was demonstrated on a robotic walking guide (Moon and Seo, 2019) where the forces exerted on the handles and the information of a depth camera were used as inputs to a neural network. Similarly, a reinforcement learning approach has also been tested to enhance force feedback to the user by providing haptic cues to guide the user hand movements (Walker et al., 2018).

To summarize, the current studies envisage ways of using force feedback and body monitoring from the user to model pHRI. Relevant outcomes suggests that force feedback can be modeled to optimize human–robot cooperative tasks by assigning active or passive roles; that the user’s trajectory and location can be partially or entirely estimated by using force feedback during cooperative tasks; and that user’s comfort and acceptance seems to increase when force feedback is integrated with artificial intelligence in assisted tasks.

### D User Modeling and Safety Assessment

In domestic applications, task-qualified engineers, who have operational knowledge of the robotic platforms, may not always be physically present. Instead, it is likely that people with a range of varying user profiles would be most frequently using or co-operating with assistive robotic systems. An understanding of user conditions, needs and limitations is therefore required for effective deployment of assistive robots, particularly to support Activities of Daily Living (ADLs) (Bedaf et al., 2015). In addition, people requiring these technologies are also likely to be supported by other people, such as their relatives, health professionals and caregivers, and as such their specific needs also have to be taken into consideration (García-Soler et al., 2018; Beer et al., 2012). This information can serve as a starting point in safe user-centred design that considers the combined needs of the all potential users of the systems.

User modeling seems to be a key aspect to address their needs, and to understand their limitations through a personalized interaction, as depicted by some publications (García-Soler et al., 2018). For instance, user modeling studies involving kinematic evaluation tests can determine the user safe workspace in the robot’s movement envelope (Zhang et al., 2019). Similarly, an estimation of the range of physical forces exerted by a modeled arm during a simulated assisted dressing task was performed by Erickson et al. (2017). Kapusta et al. (2016) also conducted kinematic studies to map the relationship between clothing and users from data-driven haptic perception modeling. Furthermore, people who need physical assistance may present personal traits such as limited mobility or special sensitivity to touch. Therefore, it would be important to tailor or personalize robot’s assistance, so that there is no unintentional exacerbation of a known user condition. Given the generic nature of current safety standards, they are unable to fully cover the high variance of the user requirements needed for designing safe assistive robots.

### E Gaps in the Current Research

The review of the literature has identified the following issues that are significant when considering safety of physically assistive robots:

• For complex, non-deterministic contexts, there are no existing hazard analysis techniques that can provide comprehensive coverage for risks associated with physically assistive robotic applications.
• Physical contact during interaction is generally regarded as a safety hazard and safety standards relate to collision avoidance, although some studies show that contact forces could be used constructively for feedback and safe operation.
A clear methodology for pHRI personalization has not been identified.

## III Robot-Assisted Dressing: pHRI Safety Analysis Case Study

The application considered in this paper is robot assisted dressing. It was developed as part of the project ‘Assistive interactive robotic system for support in dressing—I-DRESS’ (i-dress-project.iri.u*p*c.edu) funded by CHIST-ERA (chistera.eu) and EPSRC to develop a multi-modal interaction framework and intuitive user interfaces to facilitate safe physical interaction and cognitive robot behavior. The project considered proactive assistance for dressing tasks (shoes, jackets) to support people with disabilities, or high risk healthcare workers (personal protective equipment such as surgical gowns). A key objective for the project was ensuring safety by using multi-modal inputs accounting for environment dynamics, human errors and ergonomic limits. We used a Baxter robot to investigate dressing dynamics thanks to its similarity with the human arms (bi-manual operation) (Chance et al., 2016; Chance et al., 2017). To enable dressing assistance, we defined the overall system requirements taking into account a user to be dressed, a garment, and the environment (Table 1 shows those relevant to pHRI).

**TABLE 1:** System requirements.

ID	Description
UR001	The system must respond to voice commands in <1s
UR002	The system must be able to determine the task type requested
UR005	The system must be able to identify garments using sensors specified
UR006	The system must be able to grasp the garment correctly and hold it in a suitable position for dressing
UR007	The system must be able to determine handedness or orientation of the garment
UR008	The system must be able to detect the user and environment to within ±5 mm
UR009	The system must be able to detect and differentiate arms, legs and torso with onboard sensors and processing
UR010	The system must be able to move the garment toward the user
UR011	The system must be able to track the position of the target limb through small movements ±200 mm
UR012	The system must be able to detect dressing errors based on force limitations
UR013	The system must be able to initiate HRI strategies based on error detection
UR014	The system must know when a dressing task is complete
UR015	The system must contain a user profile that can be adapted during learning and operation
UR016	The system must be able to learn dressing motions in learning mode and during opetation
UR017	The system must employ a safety executive function at all times

### A Methodology

For understanding the safety requirements for a physically assistive robot to support dressing, we started with evaluating the suitability of existing hazard assessment approaches for identifying safety-related issues. We applied two hazard analysis techniques to our assistive dressing task scenario with the aim of evaluating their applicability and limitations. The results are presented in this section and a risk register which emerged from applying these techniques is presented in Supplementary Appendix. In Section IV, we analyze and discuss these results against the gaps exposed in the literature review in Section II, and against our pHRI requirements to suggest safety analysis approaches for pHRI in social care applications.

In our method, SHARD+UML (a HAZOP+UML variant) was used to assess the robot’s operational safety while STPA was used for analyzing safety-related issues resulting from human actions. The reason for choosing these techniques was not only to evaluate their suitability for our particular application, but also to consider their general applicability for similar physically assistive HRI tasks.

The method followed in this study was divided into two investigations, Section III and Section IV, for which two research parties worked separately. This is important to prevent bias between sections (e.g., Section IV affecting Section III results. In other words, once the hazard identification and risk analysis are performed by one researcher, an evaluation of the tools shortcomings was performed by a different researcher to validate the concerns exposed in the literature review.

### B IDRESS: Scenario Description

**FIGURE 2:**
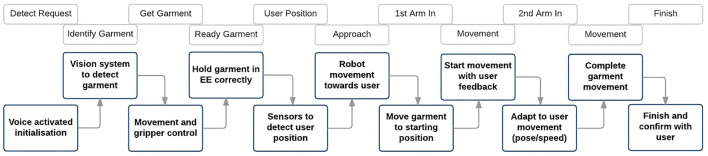
Jacket or gown dressing scenario.

In this section, we present our use case that consists of an assisted dressing task performed by a robotic platform (Baxter robot). From a systems engineering perspective, this function is achieved by the concatenation of modules that expect a series of inputs and outputs. To help a user put on a jacket or a surgical gown, we first need to confirm their willingness to participate. From then on, the robot will use different sensors such as microphones, cameras, motor currents and others to advance in an orderly fashion through the expected dressing sequence. In Figure 2, we see an example of this sequence in which after a voice confirmation, the robot detects, grasps and positions the garment so that it can be donned on the person in an ergonomically appropriate manner, ending with a user voice confirmation. This is a simplified scenario that does not consider all possible outcomes that could arise due to unexpected user actions or environmental factors at the time of execution, but defines the objective and physical contact implications.

Figure 2 shows a flow chart of one possible scenario during a jacket dressing assistance tasks. This will be useful to identify the situations in which the system must make decisions, monitor the user and handle basic errors in future sections. In this chart, all possible robot errors are not considered, and we make the assumption that the user complies with the predefined dressing sequence.

### C UML: System Software Analysis

At this stage, we will apply “Unified Modeling Language” (UML), an extensively used tool for software applications to address the thorough breakdown of possible outcomes. A UML diagram is created by defining the logical connection between blocks and the reasoning flow. Supplementary Appendix Figure 4 shows representative user related errors that may occur in different stages of a shoe fitting task. When the UML analysis is completed, the subsequent SHARD analysis (HAZOP variant) can be initialised.

### D SHARD: Robot Hazard and Error Analysis

For the hazard analysis of the robot’s operation we chose SHARD to focus on what errors the robot (not the user) can encounter. Here we examine the UML flowchart (Supplementary Appendix Figure 4) based on the logical path of actions the robot may take. At each point or node of the UML (identified with the ID: UMLXX) we apply the SHARD analysis technique.

For each node of the UML, we apply one of the guide words: Omission, Commission, Early, Late and Value, as suggested in the SHARD analysis (Pumfrey, 1999) (See Supplementary Appendix Table 2). This gives 5 possible hazards for each node for a total of 20 nodes. Every guide word analyzed against a node is referred to as an entry. For every entry, a rudimentary hazard severity level is assigned: no hazard, user annoyance, low, medium and high hazard (See Supplementary Appendix Table 2) where an “annoyance” implies user waiting and/or distracted; “low” implies a trivial injury risk (discomfort); “medium” implies a minor injury risk (bruising, abrasion); and “high” implies a serious injury risk (strain, sprain, incapacitated). This hazard severity level corresponds approximately to existing hazard severity standards such as the *Abbreviated Injury Scale (AIS)* (Loftis et al., 2018), a widely used scale of injury severity used in the automotive and medical sectors. The severity levels described in Supplementary Appendix Table 2 correspond approximately to levels 1–3 of the AIS Severity Scale.

A full list of potential hazards from this exercise can be found in Supplementary Appendix Table 5, stating the causes of the hazards and proposed design recommendations. The most relevant hazards identified in the process are related to but not limited to: communication delays or long conversation performed by the robot; not clear sensor interpretation by the robot; garment snagging errors; user distraction; second actor interventions among others.

After exploring this SHARD+UML combination, we confirmed it is good for software flow, however, some human related-aspects were superficially developed hence STPA was tested to check if more information could be retrieved.

### E STPA: User Error Analysis

STPA, or Systems-Theoretic Process Analysis, is based on systems thinking and a new model of accident causation based on systems theory rather than reliability theory. While traditional techniques were created to anticipate component failure accidents, STPA was engineered to also address increasingly common component interaction accidents arising from design flaws or unsafe interactions among non-failing (operational) components (Leveson and Thomas, 2018). For this reason, STPA is often described as a super-set of the hazards causes identified by traditional techniques. STPA was conceived to address the evolving complexity of technology from mid-20th century which led to computers and digital systems. However, we have to consider that STPA still requires to be adapted and completed to the specific task and we should expect a higher level of variables to assess compared to traditional methods (Bolbot et al., 2019).

The range of possible human actions within a given context is finite but could be numerous. We therefore use the task scenario in Section IIIB to define the essential actions and the STPA method to provide an initial abstraction of potentially unsafe behaviors.

**FIGURE 3:**
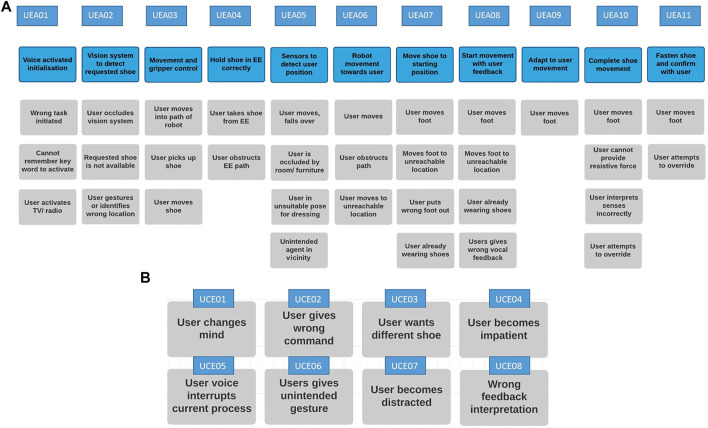
**(A)** User error analysis in a dressing scenario. **(B)** User errors common to any task scenario element.

The task scenario is segmented and shown in the top row of Figure 3A). Under each task segment, there are a few examples of the user initiating or undertaking actions that could result in a potential hazard. Each part of the task is identified with an ID starting UEAXX (for User Error Analysis) which is used for identifying hazards. This part of the hazard analysis is particularly pertinent given that this system may be used for older adults with memory or mobility issues who can also make unpredictable movements, gestures or utterances. The list of user errors attempts to cover a range of the potential issues that is enough to explore the ideas proposed in this paper.

## IV Inclusive Measurements for Safe Real World Robotics Design

In this section the results from Section III are discussed followed by pHRI design recommendations based on our use case study.

### A Hazard Identification

The hazard assessment methods exposed 41 user-error based hazards using STPA and 105 robot-error based hazards using a combination of SHARD and UML which are fully documented in Supplementary Appendix. In summary, the high and medium risks associated with the user can be summarized in three topics which also cover the low risks related to physical damage:

• Collision, friction or pulling between the user and the robot due to the user’s loss of balance or entanglement with the garment.
• Obstruction or occlusion of the robot’s trajectory by the user or by an 2nd party affecting robot performance and/or leading to collision.
• Dynamic natural character of the user actions (sudden movements, distractions, change of intention, confusion and miscommunication).

These risks also appear to be widespread across the dressing sequence meaning that, for example, the user could loss balance at different stages of the dressing task. In other words, we could also say that all examples belong to one or more of the following types:

• Wrong user actions due to mistakes or wrong/unclear information received from the robotic system.
• Invalid request performed by the robot due to the lack of knowledge about the user (e.g., user mobility or joint movement limitation).
• Timing of the collaborative actions.

This focused exposition of the hazards facilitates the understanding of the challenges that a robot handles and how they could result in the state explosion problem affecting the completeness of the technique. As an example, hazards from complex interactions that could damage external agents were not reflected in the analysis; instead, the risk assessment focuses on the user distraction and possible injuries caused by 2nd actors. This is the case of failures due to interactions with pets, relatives or other external agents in the vicinity that could result in damage to these 2nd actors inside the robot workspace. This disregard to real world applications (especially within the assistive care field) could degenerate in the state-space explosion problem for possible hazards, mainly because real world applications are less constraint than deterministic robotic technologies. Furthermore, addressing this increasing complexity could become unfeasible when applying traditional hazard identification methods as they normally have to be manually tailored by engineering experts.

In the assessment process, a type of hazard was identified and labeled as “annoyance.” This hazard occurs as a result of a bad timing, false positives, and wrong actions of a non-hazardous risk, however they can produce a delayed action, distraction, or even user’s trust loss in the technology. The “annoyance” hazard considers the user’s mood or change of mind during the task execution, and over long periods of exposure to the robot’s failures. In this case, user modeling would be required, and it should account for health care and engineering models of this hazardous phenomenon. Completeness could be compromised when the engineer alone may lack the ability to address the clinical nuances of the long-term “annoyance” risk during the hazard analysis stage.

These features still need rigorous protocols as seen in the Literature Review Section IIA) which may invalidate the sole use of traditional methodologies due to an increase in the range of variables that could break a safety clause in a real-world robotics design. Hence, traditional approaches could be combined with interdisciplinary assessments of safety and user modeling resulting in improved versions of, for instance HAZOP, STPA and HRA for the human-machine interface safety assessment.

To illustrate how modifying traditional methods could help to solve these type of issues, we propose some practical solutions. We first recommend the use of new keywords as other studies propose for performing SHARD analyses (Bolbot et al., 2019); but with a clinical tweak. For example, we suggest using a keyword that considers periodicity in SHARD (e.g., “Periodically”) or, at least, a reiteration counter for each module in the UML diagram, that could estimate the long-term harmful effects of repetitive robot action loops on the user. Therefore, this loop would be clinically assessed by a healthcare experts providing a hazard such as“excessively frequent assistance could lead to dependence.” In this solution, we propose a new management of the user modeling component for real world technologies. In other words, We raise a call for regulating the intervention of experts of different disciplines to maximize the completeness in the design of standard methods and guidelines for its management and application.

### B Safe Design Recommendations

Safe design implies awareness of possible hazard which requires understanding of all the agents involved in the scene and their interactions. In our use case, we observed a prominent presence of continuous contact, and high-risk hazards identified with collision (See UEA 03;04;06;09;10;11 and UML 19 in Supplementary Appendix Table 3 and Supplementary Appendix Table 5). In this scenario, a severity of a failure is highly dependent on the amount, concentration and duration of the pressure that is exerted on the user. Therefore, a model of this phenomenon could infer the actual force applied on the contact surfaces and identification of potential unsafe configurations. For example, model of the force exerted on the user could be used to improve the robot’s compliance in the user interaction. In fact, general models of these interactions could be retrofitted for other approaches of modeling continuous contact for assisted dressing as seen in Section IIC, Kapusta et al. (2016), Zhang et al. (2019), Erickson et al. (2017), Cirillo et al. (2016).

Certain dressing scenarios could produce hazardous situations that can become an instantaneous high risk for the user In these situations, the user could play an important role by validating the robot’s perception and supervising the robot’s behavior to alleviate risk escalation. This collaborative perspective of HRI could be used to set active and passive roles adapted to the user preferences and cues to start or follow actions in cooperation with the robot. In fact, a relevant study (Roncone et al., 2017) claims that the task efficiency increases when the user has an active role. A survey performed with older adults regarding assistive robots revealed the possibility to verify the robot’s action plan before it is executed through a display screen (García-Soler et al., 2018) was a desired attribute for most participants. Although some physical and cognitive impairments may hinder the supervision of robotic assistance, users can still contribute to safe interaction by monitoring correct functioning of the platform and providing inputs using interactive graphic displays or voice confirmation, thereby, enhancing the robot decision making in real time. Taking advantage of the physical and non-physical human–robot communication channels should be a new paradigm to mitigate uncertainty and instill trust in the use of the technology.

We propose an extension of the official standards scope regarding pHRI modeling where the safe physical continuous contact is included as a performance requirement rather than a hazard to be avoided. Contributions in this direction should be sought outside engineers remit, consulting widely the fields of social, medical and legal domains. At the moment, (ISO 13482, 2014; Haddadin et al., 2008; IEC 60601-1-11, 2015; ISO/TR 22411, 2008) treat different topics that affect assistance robotics separately when an integrated standard would be more useful. For example, the IEC standard associated with medical robots for rehabilitation, assessment, compensation or alleviation (IEC/FDIS 80601, 2019) and the ISO 9241–910 standard associated with ergonomics of human-system interaction regarding tactile interfaces (ISO 9241-910, 2011) could cross-reference (Haddadin et al., 2008) and (ISO 13482, 2014) and introduce interdisciplinary documents. This could be done by introducing topics such as the user capabilities evaluation for collaboration with robots; the impairment assessment for use of robotic assistive services, among others.

## V Conclusion

The field of physically assistive robotics aims at consolidating effective healthcare support for the growing population of older adults and professional treatment of infectious diseases. Physical contact, direct or indirect, over extended periods of time is often an essential part of the assistance (e.g., for dressing). Industry standards, intended only for manufacturing robotics applications, do not directly cover the continuous physical interaction or user modeling guidelines associated with healthcare applications. In fact, many hazard identification methods remain optimized for reactive approaches such as collision avoidance missing expansion to the high dimensionality typical of pHRI. An application example of two hazard analysis methods was conducted by one researcher to produce typical approaches to risks, and, consecutively, another researcher proceeded to assess the effectiveness of such methods. SHARD+UML and STPA were conducted and a risk register for each technique was produced. Many hazard were correctly identified but the methods could fail at fully covering action timing during user-robot collaboration; user and 2nd parties (e.g., pets or relatives) errors and behavior; and familiarity with user health profile to account for long-term effects. Finally, the importance of some safe design recommendations is presented including guidelines on continuous physical contact modeling; the encouragement of task validation through collaboration with the user, and the multidisciplinary assessment of risks and the system’s design.

## Data Availability

The original contributions presented in the study are included in the article/Supplementary Material, further inquiries can be directed to the corresponding author.

## References

[B1] Abdulkhaleq A. Baumeister M. Böhmert H. Wagner S. (2018). Missing No Interaction-Using STPA for Identifying Hazardous Interactions of Automated Driving Systems. IJSS 02 (01), 115–124. 10.24900/ijss/0201115124.2018.0301

[B2] Albu-Schäeffer A. Eiberger O. Grebenstein M. Haddadin S. Ott C. Wimböck T. (2008). Soft Robotics: From Torque Feedback Controlled Lightweight Robots to Intrinsically Compliant Systems. IEEE Robotics Automation Mag. 15, 20–30. 10.1109/iros40897.2019.8968251

[B3] Ansari R. J. Karayiannidis Y. Sjöber J. (2018). “Physical Human–Robot Interaction through a Jointly-Held Object Based on Kinesthetic Perception,” in 2018 27th IEEE International Symposium on Robot and Human Interactive Communication (RO-MAN), 1099–1104.

[B4] Bedaf S. Gelderblom G. J. de Witte L. (2015). Overview and Categorization of Robots Supporting Independent Living of Elderly People: What Activities Do They Support and How Far Have They Developed. Assistive Tech. 27 (2), 88–100. 10.1080/10400435.2014.978916 26132353

[B5] Beer J. M. Prakash A. Smarr C.-A. Mitzner T. L. Kemp C. C. Rogers W. A. (2012). "Commanding Your Robot" Older Adults' Preferences for Methods of Robot Control. Proc. Hum. Factors Ergon. Soc. Annu. Meet. 56 (1), 1263–1267. 10.1177/1071181312561224 31337947 PMC6649675

[B6] Beetz M. Bartels G. Albu-Schäffer A. Bálint-Benczédi F. Belder R. Beßler D. (2015). “Robotic Agents Capable of Natural and Safe Physical Interaction with Human Co-workers,” in 2015 IEEE/RSJ International Conference on Intelligent Robots and Systems (IROS) (IEEE), 6528–6535.

[B7] Bensaci C. Zennir Y. Pomorski D. (2018). “A Comparative Study of STPA Hierarchical Structures in Risk Analysis: The Case of a Complex Multi-Robot mobile System,” in European Conference on Electrical Engineering & Computer Science (EECS).

[B8] Bolbot V. Theotokatos G. Bujorianu L. M. Boulougouris E. Vassalos D. (2019). Vulnerabilities and Safety Assurance Methods in Cyber-Physical Systems: A Comprehensive Review. Reliability Eng. Syst. Saf. 182, 179–193. 10.1016/j.ress.2018.09.004 Available at: http://www.sciencedirect.com/science/article/pii/S0951832018302709.

[B9] BS EN 61882 (2019). BS EN 61882:2016 Hazard and Operability Studies (HAZOP Studies)—Application Guide. BSI Standards Publication, Tech. Rep.

[B10] Chance G. Camilleri A. Winstone B. Caleb-Solly P. Dogramadzi S. (2016). “An Assistive Robot to Support Dressing-Strategies for Planning and Error Handling,”in 2016 6th IEEE International Conference on Biomedical Robotics and Biomechatronics (BioRob) (IEEE), 774–780.

[B11] Chance G. Jevtić A. Caleb-Solly P. Dogramadzi S. (2017). A Quantitative Analysis of Dressing Dynamics for Robotic Dressing Assistance. Front. Robot. AI 4 (13), 121. 10.3389/frobt.2017.00013

[B12] Cirillo A. Ficuciello F. Natale C. Pirozzi S. Villani L. (2016). A Conformable Force/Tactile Skin for Physical Human–Robot Interaction. IEEE Robot. Autom. Lett. 1 (1), 41–48. 10.1109/lra.2015.2505061

[B13] De Luca A. Albu-Schaffer A. Haddadin S. Hirzinger G. (2006). “Collision Detection and Safe Reaction with the Dlr-Iii Lightweight Manipulator Arm,” in 2006 IEEE/RSJ International Conference on Intelligent Robots and Systems (IEEE), 1623–1630.

[B14] De Luca A. Flacco F. Bicchi A. Schiavi R. (2009). “Nonlinear Decoupled Motion-Stiffness Control and Collision Detection/reaction for the Vsa-Ii Variable Stiffness Device,” in 2009 IEEE/RSJ International Conference on Intelligent Robots and Systems (IEEE), 5487–5494.

[B15] De Luca A. Flacco F. (2012). “Integrated Control for Phri: Collision Avoidance, Detection, Reaction and Collaboration,” in IEEE RAS EMBS International Conference on Biomedical Robotics and Biomechatronics (BioRob), 288–295.

[B16] De Luca A. (2015). Safe and Autonomous Physical Human-Aware Robot Interaction. Project Supported by the European Commission under the 7th Framework Programme. Available at: http://www.saphari.eu/.

[B17] Do Hoang Q. A. Guiochet J. Powell D. Kaâniche M. (2012). “Human–robot Interactions: Model-Based Risk Analysis and Safety Case Construction,” in Embedded Real Time Software and Systems (ERTS2).

[B18] Eder K. Harper C. Leonards U. (2014). “Towards the Safety of Human-In-The-Loop Robotics: Challenges and Opportunities for Safety Assurance of Robotic Co-workers’,” in The 23rd IEEE International Symposium on Robot and Human Interactive Communication (IEEE), 660–665.

[B19] El Zaatari S. Marei M. Li W. Usman Z. (2019). Cobot Programming for Collaborative Industrial Tasks: An Overview. Robotics Autonomous Syst. 116, 162–180. 10.1016/j.robot.2019.03.003

[B20] Erickson Z. Clegg A. Yu W. Turk G. Liu C. K. Kemp C. C. (2017). “What Does the Person Feel? Learning to Infer Applied Forces during Robot-Assisted Dressing,” in 2017 IEEE International Conference on Robotics and Automation (ICRA), 6058–6065.

[B21] Fossum K. Danielsen B.-E. Aarseth W. Johnsen S. (2018). A Project Management Issue of New Technology Developments: A Case Study on Lack of Human Factors’ Attention in Human–Robot Interaction. Proc. Inst. Mech. Eng. O: J. Risk Reliability 232, 164–173. 10.1177/1748006x17728601

[B22] García-Soler Á. Facal D. Díaz-Orueta U. Pigini L. Blasi L. Qiu R. (2018). Inclusion of Service Robots in the Daily Lives of Frail Older Users: A Step-by-step Definition Procedure on Users' Requirements. Arch. Gerontol. Geriatr. 74, 191–196. 10.1016/j.archger.2017.10.024 29128788

[B23] Gienger M. Ruiken D. Bates T. Regaieg M. MeiBner M. Kober J. Seiwald P. Hildebrandt A. (2018). ““Human–robot Cooperative Object Manipulation with Contact Changes,” in IEEE/RSJ International Conference on Intelligent Robots and Systems (IROS), 1354–1360.

[B24] Guiochet J. Do Hoang Q. A. Kaâniche M. Powell D. (2012). “Applying Existing Standards to a Medical Rehabilitation Robot: Limits and Challenges,” in IEEE/RSJ International Conference on Intelligent Robots and Systems (IROS 2012).

[B25] Guiochet J. (2016). Hazard Analysis of Human–Robot Interactions with HAZOP-UML. Saf. Sci. 84, 225–237. 10.1016/j.ssci.2015.12.017

[B26] Guiochet J. Machin M. Waeselynck H. (2017). Safety-critical Advanced Robots: A Survey. Robotics Autonomous Syst. 94, 43–52. 10.1016/j.robot.2017.04.004

[B27] Haddadin S. Albu-Schäffer A. Hirzinger G. (2009). Requirements for Safe Robots: Measurements, Analysis and New Insights. Int. J. Robotics Res. 28 (11-12), 1507–1527. 10.1177/0278364909343970

[B28] IEC 31010 (2019). IEC 31010:2019 Risk Management. Risk Assessment Techniques. BSI Standards Publication, Tech. Rep.

[B29] IEC 60601-1-11 (2015). IEC 60601-1-11:2015 - Medical Electrical Equipment—Part 1-11: General Requirements for Basic Safety and Essential Performance—Collateral Standard: Requirements for Medical Electrical Equipment and Medical Electrical Systems Used in the home Healthcare Environment. BSI Standards Publication, Tech. Rep.

[B30] IEC 60812 (2018). IEC 60812:2018 - Failure Modes and Effects Analysis (Fmea and Fmeca). BSI Standards Publication, Tech. Rep.

[B31] IEC/FDIS 80601 (2019). IEC/FDIS 80601-2-78 Medical Electrical Equipment—Part 2-78: Particular Requirements for Basic Safety and Essential Performance of Medical Robots for Rehabilitation, Assessment, Compensation or Alleviation. ISO, Tech. Rep.

[B32] ISO 13482 (2014). ISO 13482:2014 Robots and Robotic Devices—Safety Requirements for Personal Care Robots. ISO-IEC, Tech. Rep.

[B33] ISO 9241-910 (2011). ISO 9241-910:2011 - Ergonomics of Human-System Interaction—Part 910: Framework for Tactile and Haptic Interaction. BSI Standards Publication, Tech. Rep.

[B34] ISO/TR 22411 (2008). ISO/TR 22411:2008 Ergonomics Data and Guidelines for the Application of ISO/IEC Guide 71 to Products and Services to Address the Needs of Older Persons and Persons with Disabilities. ISO-IEC, Tech. Rep.

[B35] Kapusta A. Yu W. Bhattacharjee T. Liu C. K. Turk G. Kemp C. C. (2016). “Data-driven Haptic Perception for Robot-Assisted Dressing,” in 2016 25th IEEE international symposium on robot and human interactive communication (RO-MAN) IEEE, 451–458.

[B36] Kucukyilmaz A. Demiris Y. (2018). Learning Shared Control by Demonstration for Personalized Wheelchair Assistance. IEEE Trans. Haptics 11 (3), 431–442. 10.1109/toh.2018.2804911 29994370

[B37] Leveson N. (2004). A New Accident Model for Engineering Safer Systems. Saf. Sci. 42 (4), 237–270. 10.1016/s0925-7535(03)00047-x

[B38] Leveson N. Thomas J. (2018). STPA Handbook. MIT Partnership For Systems Approaches To Safety And Security (PSASS). Available at: http://psas.scripts.mit.edu/home/materials/

[B39] Loftis K. L. Price J. Gillich P. J. (2018). Evolution of the Abbreviated Injury Scale: 1990-2015, Traffic Inj. Prev. 19. sup2, S109–S113. 10.1080/15389588.2018.1512747 30543458

[B40] Moon H.-S. Seo J. (2019). “Prediction of Human Trajectory Following a Haptic Robotic Guide Using Recurrent Neural Networks,” in 2019 IEEE World Haptics Conference (WHC) (IEEE), 157–162.

[B41] Mörtl A. Lawitzky M. Kucukyilmaz A. Sezgin M. Basdogan C. Hirche S. (2012). The Role of Roles: Physical Cooperation between Humans and Robots. Int. J. Robotics Res. 31 (13), 1656–1674. 10.1177/0278364912455366

[B42] Salem M. Lakatos G. Amirabdollahian F. Dautenhahn K.. (2015). “Towards Safe and Trustworthy Social Robots: Ethical Challenges and Practical Issues,” in International Conference on Social Robotics.Berlin: Springer, pp. 584–593. 10.1007/978-3-319-25554-5_58

[B43] Nishimura R. Wada T. Sugiyama S. (2015). Haptic Shared Control in Steering Operation Based on Cooperative Status between a Driver and a Driver Assistance System. J. Human–Robot Interaction 4 (3), 19–37. 10.5898/4.3.nishimura

[B44] Onofrio R. Trucco P. (2018). Human Reliability Analysis (Hra) in Surgery: Identification and Assessment of Influencing Factors. Saf. Sci. 110, 110–123. 10.1016/j.ssci.2018.08.004

[B45] Pumfrey D. J. (1999). The Principled Design of Computer System Safety Analyses. University of York. Ph.D. dissertation.

[B46] Roncone A. Mangin O. Scassellati B. (2017). “Transparent Role Assignment and Task Allocation in Human Robot Collaboration,”in 2017 IEEE International Conference on Robotics and Automation (ICRA) IEEE, 1014–1021.

[B47] Haddadin S. Albu-Schaffer A. De Luca A. Hirzinger G. (2008). “Collision Detection and Reaction: A Contribution to Safe Physical Human–Robot Interaction,” in IEEE/RSJ International Conference on Intelligent Robots and Systems, pp. 3356–3363.

[B48] Takagi A. Usai F. Ganesh G. Sanguineti V. Burdet E. (2018). Haptic Communication between Humans Is Tuned by the Hard or Soft Mechanics of Interaction. Plos Comput. Biol. 14 (3), e1005971–17. 10.1371/journal.pcbi.1005971 29565966 PMC5863953

[B49] Walker J. M. Okamura A. M. Kochenderfer M. J. (2018). “Gaussian Process Dynamic Programming for Optimizing Ungrounded Haptic Guidance,” in 2018 IEEE/RSJ International Conference on Intelligent Robots and Systems (IROS) (IEEE), 8758–8764.

[B50] Walther S. Guhl T. (2014). “Classification of Physical Human–Robot Interaction Scenarios to Identify Relevant Requirements,” in ISR/Robotik 41st International Symposium on Robotics (VDE), 1–8.

[B51] Zafrani O. Nimrod G. (2019). Towards a Holistic Approach to Studying Human–Robot Interaction in Later Life. The Gerontologist 59 (1), e26–e36. 10.1093/geront/gny077 30016437

[B52] Zhang F. Cully A. Demiris Y. (2019). Probabilistic Real-Time User Posture Tracking for Personalized Robot-Assisted Dressing. IEEE Trans. Robotics 14, 1–16. 10.1109/tro.2019.2904461

[B53] Zube A. (2015). “Combined Workspace Monitoring and Collision Avoidance for mobile Manipulators,,” in IEEE 20th Conference on Emerging Technologies and Factory Automation (ETFA) (IEEE), 1–8.

