# Supplementary material for: Safety Assessment Review of a Dressing Assistance Robot
Source: Front Robot AI. 2021 Jun 14;8:667316. doi: 10.3389/frobt.2021.667316 (PMC8236579; doi:10.3389/frobt.2021.667316)
Supplement: Supplementary file 3 [file DataSheet1.pdf]

VII. Appendix

Appendix A):

For each node of the UML we apply each of the keywords shown in Table 2 (upper), as suggested in the SHARD analysis . This gives 5 possible hazards for each node for a total of 20 nodes. Every guide word analysed against a node is referred to as an entry. For every entry a rudimentary hazard level is assigned, see Table 2 (lower), to raise awareness of the most important hazards to the reader.

Guide Word	Definition
Omission	The service is never delivered, i. e. there is no communication
Commission	A service is delivered when not required, i. e. there is an unexpected communication
Early	The service (communication) occurs earlier than intended
Late	The service (communication) occurs later than intended
Value	The information (data) delivered has the wrong value

Hazard Severity Level	Description
no hazard	No safety related consequences
annoyance	No safety related consequences, but a loss of service performance that may cause annoyance (e.g. user waiting and becomes distracted)
low	Minor injury (discomfort, bruising)
medium	Moderate injury (small cut, abrasion)
high	Serious injury (strain, sprain, incapacitated)

Table 2: Guide words (upper) and hazard levels (lower) used in the analysis

Appendix B)

The full human error analysis for the garment dressing scenario is shown in Table 3 below. This table considered errors specific to the task segment as shown in Figure 3 a)

Task	Index	Description	Possible Causes	Effects	Detection & Protection	Justification or Design Recommendation	Hazard Level
UEA 01	S 01	Wrong task initiated	User said the wrong thing	Wrong task may be initiated	Take cues from other modalities	Use multimodal input for high probability Implement HRI if in doubt	medium
UEA 01	S 02	Forget key words	User memory issues	Task not started	Take cues from other modalities	Use multimodal input for high probability Implement HRI if in doubt	annoyance
UEA 01	S 03	Background noise interference	TV or radio on, unintended agent	Incorrect interpretation, unexpected robot behaviour	Check initiation of task with other modalities	Use multimodal input for high probability Implement HRI if in doubt	medium
UEA 02	S 04	User occludes visual system	User wanders in front of robot	User tracking lost	System must stop any movement	Regain tracking or implement HRI	low
UEA 02	S 05	Shoe/jacket not available	User picked or moved shoe	Garment tracking lost	Use proximity sensors, tell user to not occlude/ pick up garment or move around	Ask user where garment is	no hazard
UEA 02	S 06	Wrong gesture given	User confusion	Incorrect interpretation	Compare user intention from other modalities	Ask user to confirm if ambiguity exists not resolvable by multimodal inputs	low
UEA 03	S 07	User collision course	User loses balance	User at risk of injury	Offer balance support or reminder to be seated	Safety proximity sensors, compliance mode during all movement	high
UEA 03	S 08	Shoe/jacket not available	User picks up shoe/jacket	Task not started or pauses	Use proximity sensors, tell user to not occlude/ pick up garment or move around	Ask user to confirm if ambiguity exists not resolvable by multimodal inputs	annoyance
UEA 03	S 09	User moves shoe/jacket	User believes they are helping	Task segmentation lost	Use proximity sensors, tell user to not occlude/ pick up garment or move around	Ask user to remain still, confirm task segment through HRI if necessary	low
UEA 04	S 10	User takes shoe/jacket from EE	User pulls out of EE grip	EE moves with empty grip	Use proximity sensors, tell user to not occlude/ pick up garment or move around	Ask user to confirm if ambiguity exists not resolvable by multimodal inputs	low
UEA 04	S 11	User obstructs EE path	User wanders in front of robot	Task not started, potential collision with EE	Use proximity sensors, tell user to not occlude/ pick up garment or move around	Robot pauses if user moves when in close proximity	high
UEA 05	S 12	User moves	User loses balance	User at risk of injury	Use proximity sensors, tell user to not occlude/ pick up garment or move around	Promt user to remain still at certain points and especially during movement	medium
UEA 05	S 13	User occluded	User moves behind environment article	User tracking lost	Use proximity sensors, tell user to not occlude/ pick up garment or move around	If user tracking is lost use speech HRI	no hazard
UEA 05	S 14	Unsuitable pose	User does not understand workspace limits	Task not started or pauses	Use proximity sensors, tell user to not occlude/ pick up garment or move around	Use speech based HRI to get user into more appropriate pose	no hazard
UEA 05	S 15	Unintended agent	2nd actor enters stage	System tracks 2nd actor, system receives speech from 2nd actor, task not started, pauses	Ask if there is a 2nd actor or noise interference	Ask if there is a 2nd person in the room	no hazard
UEA 06	S 16	User moves	User loses balance, believe they are assisting	User tracking lost user injury	Tell user to not occlude/ pick up garment or move around	Implement a non-rigid trajectory that can track the user movement during dressing	medium
UEA 06	S 17	User obstructs path	User wanders in front of robot	User at risk of injury/collision task interrupted	Use proximity sensors, tell user to not occlude/ pick up garment or move around	Track user position and define boundary where user is alerted (alarm) and/or define an emergency stop procedure	high
UEA 06	S 18	User out of range	User wanders off	User tracking lost, task interrupted	Use proximity sensors, tell user to remain within range and in certain pose	Track user position and define boundary where user is alerted out of functioning range of the robot	no hazard
UEA 07	S 19	User moves/ changes pose	User loses balance, believe they are assisting	User tracking lost, task interrupted	Use proximity sensors, tell user to not occlude/ pick up garment or move around	Robot must adapt to changes in user position, consideration to how the user may help the robot too	medium

UEA 07	S 20	Foot/Arm out of range	User does not understand workspace limits	User tracking lost, task interrupted	Use proximity sensors, tell user to remain within range and in certain pose	Track user position and define boundary where user is alerted out of functioning range of the robot	low
UEA 07	S 21	Wrong foot/arm presented	Interpretation incorrect/confusion	Incorrect user interpretation, garment incorrectly dressed	Pose detection	Use multimodal input for high probability Implement HRI if in doubt	low
UEA 07	S 22	User already wearing garment	User confusion	User struggles dressing, High force experienced	Visual recognition system or current user garment	Garment detection of existing clothing may be difficult and out of scope, suggest using HRI to confirm if there is doubt	medium
UEA 08	S 23	User moves/ changes pose	User loses balance, believe they are assisting	Potential injury, task interrupted	Use proximity sensors, tell user to not occlude/ pick up garment or move around	Robot must adapt to changes in user position, consideration to how the user may help the robot too	high
UEA 08	S 24	Foot/Arm out of range	User does not understand workspace limits	Task interrupted	Tell user to remain within range and in certain pose	Remind user they are out of range, show picture or project boundary onto the floor (e.g. laser line)	annoyance
UEA 08	S 25	User already wearing garment	User confusion	User struggles dressing, High force experienced	Visual recognition system or current user garment	Garment detection of existing clothing may be difficult and out of scope, suggest using HRI to confirm if there is doubt	medium
UEA 08	S 26	Wrong feedback given	User confusion	Incorrect interpretation	Compare inputs from other modalities	Use multimodal input for high probability Implement HRI if in doubt	medium
UEA 09	S 27	User moves/ changes pose	User loses balance, believe they are assisting	User at risk of injury/collision task interrupted	Use proximity sensors, tell user to not occlude/ pick up garment or move around	Robot must adapt to changes in user position, consideration to how the user may help the robot too	high
UEA 10	S 28	User moves/ changes pose	User loses balance, believe they are assisting	User at risk of injury/collision task interrupted	Use proximity sensors, tell user to not occlude/ pick up garment or move around	Robot must adapt to changes in user position, consideration to how the user may help the robot too	high
UEA 10	S 29	User force too low	User mobility issues	Task cannot be completed	Use force detection	Use a force profile and lookup from user profile, if user force is too low may have to find external help (call someone)	low
UEA 10	S 30	User interpretation incorrect	User confusion	Incorrect interpretation	Compare inputs from other modalities	Use multimodal input for high probability Implement HRI if in doubt	medium
UEA 10	S 31	User override attempt	User believes they are helping	User tracking lost, Garment tracking lost, System sequence disordered	Compare inputs from other modalities	Use multimodal input for high probability Implement HRI if in doubt	medium
UEA 11	S 32	User moves/ changes pose	User loses balance, believe they are assisting	User at risk of injury/collision task interrupted	Use proximity sensors, tell user to not occlude/ pick up garment or move around	Robot must adapt to changes in user position, consideration to how the user may help the robot too	high
UEA 11	S 32	User override attempt	User believes they are helping	User tracking lost, Garment tracking lost, System sequence disordered	Compare inputs from other modalities	Use multimodal input for high probability Implement HRI if in doubt	medium

Table 3: Full hazard analysis results for human error in a specific task segment.

Appendix C)

The human hazard analysis for the garment dressing scenario is shown in Table 4 below. For this analysis For this analysis the hazards could occur in ant segment of the task as shown in Figure 3 b).

Task	Index	Description	Possible Causes	Effects	Detection & Protection	Justification or Design Recommendation	Hazard Level
UEA 01	C 01	User changes mind	User updates decision based on new information or change of plan	User jumps to different task or segment of the task or task must stop	Use HRI and multimodal user intention module	Use multimodal input for high probability<br>Implement HRI if in doubt	medium
UEA 02	C 02	User gives wrong command	User mis-spoke or was distracted	Task may procede to a different segment that the user intended	Confirm command input with user through HRI	All commands to be reviewed bu user if they are of significant potential hazard, otherwise no action required	medium
UEA 03	C 03	User wants different garment/shoe	User indicated wrong garment or changes mind	User may get the wrong garment or fitted incorrectly	Tracking or Left and Right shoe types or garment type and be able to restart dressing sequence if requested	Exit and restart dressing sequence with different garment required	no hazard
UEA 04	C 04	User becomes impatient	User personality	System less likely to be used	Perform actions in timely manner	Ensure robot can act with suitable response times	annoyance
UEA 05	C 05	User voice interrupts current process	User personality to talk over others	User intention is misrepresented or system does not hear the recent request	Listen for user voice even if talking	Use of bidirectional communication or robot always stops talking if user interrupts	no hazard
UEA 06	C 06	User gives unintended gesture	User is distracted, 2nd actor	May give unintended user intention	Confirm gesture action if score low probability	Use probability weighting on single modality inputs	low
UEA 07	C 07	User becomes distracted	External stimulii	User may collide with robot if moving	Monitor user attention	User attention should have direct interface with safety critical system responses	high
UEA 08	C 08	User gives the wrong feedback to the robot	Robot communication not clear	User may collide with robot if moving, get into the wrong task segment by misinterpreting the robots command	Give clear commands and supplement with on screen display where appropriate	Give mulitiple outputs to user when communicating	medium

Table 4: Full hazard analysis results for human error common to any task segment.

Appendix D)

The robot hazard analysis for the garment dressing scenario is shown in Table 5 below. For this analysis the hazards are mapped onto the system UML diagram shown in Figure 4

UML Task	Index	Guide Word	Deviation	Possible Causes	Effects	Detection & Protection	Justification or Design Recommendation	Hazard Level
UML 01	R 01	Omission	Request not received	- Microphone not working<br>- Activation word not in vocabulary<br>- Voice not loud enough	Dressing task not initiated	User testing	- good microphone<br>- good speech API<br>- directional microphone	no hazard
UML 01	R 02	Commission	Request starts without authorisation	Background noise or voice misinterpreted	Task initiated		2 stage task initiation verification	annoyance
UML 01	R 03	Early	n/a	n/a	n/a	n/a	n/a	no hazard
UML 01	R 04	Late	Request takes long to process/ respond	CPU power or poor coding	User annoyance/ distraction	Timer checks in code and hardware testing	Implement timer checks in code to prevent long/infinite loops	annoyance
UML 01	R 05	Value	Wrong garment is inferred by robot	Interpretation of spoken words	Stuck in loop, robot may be moving while searching	Timer checks, implement user interaction	- Implement timer checks in code to prevent long/infinite loops<br>- Interact with user if task is taking long	annoyance
UML 02	R 06	Omission	Process will halt	- garment not in visual vocabulary<br>- cannot find garment	Dressing task will interupt	Timer checks, implement user interaction	- check for long/infinit loop tasks<br>- Implement HRI assistance	annoyance
UML 02	R 07	Commission	Robot will look for garment without user request	Wrong interpretation of user voice or background noise	Robot may start moving withoutuser's request/ knowledge	user request flag	2 stage flag verification of user request	low
UML 02	R 08	Early	n/a	n/a	n/a	n/a	n/a	no hazard
UML 02	R 09	Late	Request takes long to process/ respond	CPU power or poor coding	User could become distracted and forget robot is moving	Timer checks in code, announce to user when moving	- Sounder when robot is moving<br>- reminder to user that the robot is undertaking a specific task	annoyance
UML 02	R 10	Value	Robot doesn't look for garment or looks for non-requested garment	- Process flow from request not complete<br>- Interpretation of user request	- robot may fetch wrong garment<br>- robot may hang	User interaction	Implement HRI for garment verification with picture on screen/ or spoken	annoyance
UML 03	R 11	Omission	Robot never finds garment	Vision sensors issue, obstruction, item does not exist	user waiting	watchdog timer	- Implement timer checks in code to prevent long/infinite loops<br>- Interact with user if task is taking long	annoyance
UML 03	R 12	Commission	Robot finds item incorrectly (logic error)	logic/ programming fault, software bug	robot will fetch item not there	check logic with other modalities, use HRI	2 stage task initiation verification	no hazard
UML 03	R 13	Early	Systems gets valid logic on node prior to completions of previous node	logic/ programming fault, software bug	robot will fetch item before request validated	check logic with other modalities, use HRI	2 stage task initiation verification	no hazard
UML 03	R 14	Late	System delay in getting confirmation	CPU overloaded, sensor latency	user waiting	watchdog timer	- Implement timer checks in code to prevent long/infinite loops<br>- Interact with user if task is taking long	annoyance
UML 03	R 15	Value	Robot finds item incorrectly	logic/ programming fault, software bug	robot will fetch item not there	check logic with other modalities, use HRI	2 stage task initiation verification	no hazard
UML 04	R 16	Omission	Garment not picked up	robot/motor fault, cannot plan movement, sensor failure	user may get distracted/irritated	timer/HRI	- Implement timer checks in code to prevent long/infinite loops<br>- Interact with user if task is taking long	annoyance

UML 04	R 17	Commission	Garment picked up without request made	logic failure, sensor failure/ interpretation error	user not expecting movement	Use HRI before movement	2 stage task initiation verification	high
UML 04	R 18	Early	Garment picked up prior to request validated	logic failure, sensor failure/ interpretation error	user not expecting movement	Use HRI before movement	2 stage task initiation verification	high
UML 04	R 19	Late	Garment picked up after request made (delay)	CPU overloaded, sensor latency	user may get distracted		- Implement timer checks in code to prevent long/infinite loops<br>- Interact with user if task is taking long	annoyance
UML 04	R 20	Value	Wrong movement, wrong garment	logic failure, sensor failure/ interpretation error	robot may move EE into path of user	Use HRI before movement	2 stage task initiation verification	high
UML 05	R 21	Omission	n/a	n/a	n/a	n/a	n/a	n/a
UML 05	R 22	Commission	n/a	n/a	n/a	n/a	n/a	n/a
UML 05	R 23	Early	n/a	n/a	n/a	n/a	n/a	n/a
UML 05	R 24	Late	n/a	n/a	n/a	n/a	n/a	n/a
UML 05	R 25	Value	n/a	n/a	n/a	n/a	n/a	n/a
UML 06	R 26	Omission	n/a	n/a	n/a	n/a	n/a	n/a
UML 06	R 27	Commission	n/a	n/a	n/a	n/a	n/a	n/a
UML 06	R 28	Early	n/a	n/a	n/a	n/a	n/a	n/a
UML 06	R 29	Late	n/a	n/a	n/a	n/a	n/a	n/a
UML 06	R 30	Value	n/a	n/a	n/a	n/a	n/a	n/a
UML 07	R 31	Omission	n/a	n/a	n/a	n/a	n/a	n/a
UML 07	R 32	Commission	n/a	n/a	n/a	n/a	n/a	n/a
UML 07	R 33	Early	n/a	n/a	n/a	n/a	n/a	n/a
UML 07	R 34	Late	n/a	n/a	n/a	n/a	n/a	n/a
UML 07	R 35	Value	n/a	n/a	n/a	n/a	n/a	n/a
UML 08	R 36	Omission	Process will not start as user not detected	Proximity sensor failure, detection module failure	User not detected	Sensor testing in suitable environments	Use multiple sensors or multimodal	no hazard
UML 08	R 37	Commission	Process will continue when user is not there	Proximity sensor failure, detection module failure	User Detected when not there	Sensor testing in suitable environments	Use multiple sensors or multimodal	low
UML 08	R 38	Early	n/a	-	-	-	-	no hazard
UML 08	R 39	Late	Pose detection starts late	CPU overloaded, sensor latency	user has to wait longer for pose detection to start	multiple testing	watchdog timers	annoyance
UML 08	R 40	Value	Binary output, cannot vary	-	-	-	-	no hazard
UML 09	R 41	Omission	User pose not detected	Sensor failure	Possible halt to process	multiple testing	Use multiple sensors or multimodal, implement HRI on timer	annoyance
UML 09	R 42	Commission	User pose found but invalid	Sensor failure, image distortion	Trajectory planning will use invalid information	multiple testing, multiple inputs	Use multiple sensors or multimodal, implement HRI on timer	high
UML 09	R 43	Early	n/a	-	-			no hazard
UML 09	R 44	Late	Pose detected late	CPU overloaded, sensor latency	User waiting	set time limits to maximum wait period	watchdog timers	annoyance
UML 09	R 45	Value	Wrong pose detected	Sensor failure, image distortion	Trajectory planning will use invalid information	multiple testing, multiple inputs	Use multiple sensors or multimodal, implement HRI on timer	high
UML 10	R 46	Omission	User pose not detected	Sensor failure	Possible halt to process	multiple testing	Use multiple sensors or multimodal, implement HRI on timer	annoyance
UML 10	R 47	Commission	User pose found but invalid	Sensor failure, image distortion	Trajectory planning will use invalid information	multiple testing, multiple inputs	Use multiple sensors or multimodal, implement HRI on timer	high
UML 10	R 48	Early	n/a	-	-			no hazard
UML 10	R 49	Late	Pose detected late	CPU overloaded, sensor latency	User waiting	set time limits to maximum wait period	watchdog timers	annoyance
UML 10	R 50	Value	Wrong pose detected	Sensor failure, image distortion	Trajectory planning will use invalid information	multiple testing, multiple inputs	Use multiple sensors or multimodal, implement HRI on timer	high
UML 11	R 51	Omission	Trajectory cannot be planned	Pose data never arrived	User is waiting	timer	watchdog timers	annoyance
UML 11	R 52	Commission	Trajectory data obtained but may be invalid or false	False pose information received from 2nd actor or environmnet	Robot may collide with user	additional sensors	Must validated pose through multimodal or HRI interaction	high

UML 11	R 53	Early	Trajectory data obtained but may be invalid or false on wrong pose information	False pose information received from 2nd actor or environmnet	Robot may collide with user	additional sensors	Must validated pose through multimodal or HRI interaction	high
UML 11	R 54	Late	Trajectory information takes a long time to be received	CPU overloaded, sensor latency	User is waiting	timer	watchdog timers	annoyance
UML 11	R 55	Value	The wrong trajectory is planned	False pose information received from 2nd actor or environmnet	Robot may collide with user	additional sensors	Must validated pose through multimodal or HRI interaction	high
UML 12	R 56	Omission	Trajectory cannot be planned	Pose data never arrived	User is waiting	timer	watchdog timers	annoyance
UML 12	R 57	Commission	Trajectory data obtained but may be invalid or false	False pose information received from 2nd actor or environmnet	Robot may collide with user	additional sensors	Must validated pose through multimodal or HRI interaction	high
UML 12	R 58	Early	Trajectory data obtained but may be invalid or false on wrong pose information	False pose information received from 2nd actor or environmnet	Robot may collide with user	additional sensors	Must validated pose through multimodal or HRI interaction	high
UML 12	R 59	Late	Trajectory information takes a long time to be received	CPU overloaded, sensor latency	User is waiting	timer	watchdog timers	annoyance
UML 12	R 60	Value	The wrong trajectory is planned	False pose information received from 2nd actor or environmnet	Robot may collide with user	additional sensors	Must validated pose through multimodal or HRI interaction	high
UML 13	R 61	Omission	Garment is not moved	Robot jammed, garment stuck, safety lock out	User waits	information flow check	watchdog timers	annoyance
UML 13	R 62	Commission	Garment moves with unexpected timing	False modality input(s)	Garment starts moving when user does not expect it	Movement required HRI confimation	Safety checking on modality inputs prior to any movement	high
UML 13	R 63	Early	Garment moves before trajectory is valid	False modality input(s)	Garment starts moving when user does not expect it	Movement required HRI confimation	Safety checking on modality inputs prior to any movement	high
UML 13	R 64	Late	Garment moves but after a period of delay	CPU load causes delay	User waits then garment starts moving	User may start to move and then collide when robot starts moving again	Safety checking on modality inputs prior to any movement	medium
UML 13	R 65	Value	Garment moves in wrong direction or other movement error	False modality input(s)	Movement is unexpected	Movement required HRI confimation	Safety checking on modality inputs prior to any movement	high
UML 14	R 66	Omission	Fault not detected	faulty sensor(s), incorrect sensor interpretation	Robot continues without notification of fault	HRI or learning	System should be sensitive to single modality errors until it can adapt to user	high
UML 14	R 67	Commission	Fault detected (false positive)	unexpected sensor input	Fault process enacted when there is no fault	Robot may stop or move slowly when it thinks there is an error	False positives may be the result of unexpected user movements, a learning profile may help mitigate	annoyance
UML 14	R 68	Early	Fault detected (false positive)	n/a	n/a	n/a	n/a	no hazard
UML 14	R 69	Late	Fault detected (data arrives late)	CPU overloaded, sensor latency	Fault acted upon with delay	User may struggle while system responds, catch with HRI or other modality	Must check errors through multimodal or HRI interaction	high
UML 14	R 70	Value	Wrong fault detected	faulty sensor(s), incorrect sensor interpretation	Wrong fault correction initiated	Robot performs wrong correction procedure	Must check errors through multimodal or HRI interaction	medium
UML 15	R 71	Omission	HRI request ignored	User not heard (speech)	Robot may continue to enact on previous command ignoring user	Give user feedback on requests made	Make inputs sensitive, user to wear headset to remove b/g noise	medium

UML 15	R 72	Commission	HRI request initiated (false request)	Wrong interpretation of background noise or 2nd actor	Robot may change behaviour (unexpected)	Give user feedback on requests made	Robot to stop and ask question	annoyance
UML 15	R 73	Early	HRI request initiated (false request)	Wrong interpretation of background noise or 2nd actor	Robot may change behaviour (unexpected)	Give user feedback on requests made	Robot to stop and ask question	annoyance
UML 15	R 74	Late	HRI request received late	CPU overloaded, sensor latency	Robot may not change behaviour as expected by user	Give user feedback on requests made	Check with user before performing potentially harmful actions	high
UML 15	R 75	Value	Wrong HRI interpretation	Speech interpretation module failure, gesture recognition failure	Robot will perform unexpected action	Give user feedback on requests made	Check with user before performing potentially harmful actions	high
UML 16	R 76	Omission	Roboto continues to dress after completion	Wrong trajectory, faulty sensor(s), wrong sensor interpretation	May result in excessive force	Multimodal inputs to check completion	Check finish point using multiple inputs where possible, use HRI if doubtful	high
UML 16	R 77	Commission	Dressing finishes early	Wrong trajectory, faulty sensor(s), wrong sensor interpretation	garment not on user properly, e.g. user may lose balance with shoe improperly fitted	Multimodal inputs to check completion	Check finish point using multiple inputs where possible, use HRI if doubtful	low
UML 16	R 78	Early	Dressing finishes early	Wrong trajectory, faulty sensor(s), wrong sensor interpretation	garment not on user properly	Multimodal inputs to check completion	Check finish point using multiple inputs where possible, use HRI if doubtful	low
UML 16	R 79	Late	Robot still operating but user believes dressing is complete	Robot continues to dress after completion	May result in excessive force	Multimodal inputs to check completion	Check finish point using multiple inputs where possible, use HRI if doubtful	high
UML 16	R 80	Value	n/a binary output	n/a	n/a	n/a	n/a	no hazard
UML 17	R 81	Omission	Robot does not detect garment snagging	Wrong force tolerance, sensor failure	Robot keeps moving with snagging error	Low sensor thresholds for snagging	Use low threshold values until stable learning data collected for user	high
UML 17	R 82	Commission	Robot falsly detects garment snagging	Faulty sensor(s), wrong sensor interpretation	Robot stops and asks question about snagging when there is no issue	Create learning dataset for errors to reduce false positives	Add error conditions to user profile	no hazard
UML 17	R 83	Early	n/a	n/a	n/a	n/a	n/a	no hazard
UML 17	R 84	Late	Robot does not detect garment snagging in time	Faulty sensor(s), wrong sensor interpretation	Robot keeps moving with snagging error	Low sensor thresholds for snagging	Give priority to error detection in software architecture	high
UML 17	R 85	Value	n/a	n/a	n/a	n/a	n/a	no hazard
UML 18	R 86	Omission	System does not recognise the need for new trajectory	Pose data never arrived	User is waiting	timer	watchdog timers	annoyance
UML 18	R 87	Commission	System believes need for new trajectory (falsely)	False pose information received from 2nd actor or environmnet	Robot may collide with user	additional sensors	Must validated pose through multimodal or HRI interaction	high
UML 18	R 88	Early	New trajectory requested before pose is determined	False pose information received from 2nd actor or environmnet	Robot may collide with user	additional sensors	Must validated pose through multimodal or HRI interaction	high
UML 18	R 89	Late	Trajectory information takes a long time to be received	CPU overloaded, sensor latency	User is waiting	timer	watchdog timers	annoyance
UML 18	R 90	Value	The wrong trajectory is planned	False pose information received from 2nd actor or environmnet	Robot may collide with user	additional sensors	Must validated pose through multimodal or HRI interaction	high
UML 19	R 91	Omission	System does not recognise the need for new pose	Sensor failure	Possible halt to process	multiple testing	Use multiple sensors or multimodal, implement HRI on timer	annoyance
UML 19	R 92	Commission	System believes new user pose needed	Sensor failure, image distortion	Trajectory planning will use invalid information, potential collision	multiple testing, multiple inputs	Use multiple sensors or multimodal, implement HRI on timer	high
UML 19	R 93	Early	System believes new user pose needed	Sensor failure, image distortion	Trajectory planning will use invalid	multiple testing,	Use multiple sensors or multimodal, implement HRI on timer	no hazard

					information, potential collision	multiple inputs		
UML 19	R 94	Late	System believes new user pose needed (late)	CPU overloaded, sensor latency	User waiting	set time limits to maximum wait period	watchdog timers	annoyance
UML 19	R 95	Value	Falsely believes user is in correct pose	Sensor failure, image distortion	Trajectory planning will use invalid information, potential collision	multiple testing, multiple inputs	Use multiple sensors or multimodal, implement HRI on timer	high
UML 20	R 96	Omission	Stops the user interaction/HRI	Sesnor failure, incorrect interpretation	User confused, HRI action not complete	Multimodal	Check user attention and intention through multimodal inputs	low
UML 20	R 97	Commission	The robot stops HRI mid-process	Sesnor failure, incorrect interpretation	User confused, HRI action not complete	Multimodal	Check user attention and intention through multimodal inputs	low
UML 20	R 98	Early	n/a	n/a	n/a	n/a	n/a	no hazard
UML 20	R 99	Late	Robot HRI interaction slow to start	CPU overloaded, sensor latency	User distracted, actions not taken quickly enough	Multimodal	Check user attention and intention through multimodal inputs	low
UML 20	R 100	Value	The HRI interface does not start	Sesnor failure, incorrect interpretation	HRI interaction does not start	Multimodal	Check user attention and intention through multimodal inputs	low
UML 21	R 101	Omission	Robot does not perform trajectory correction	Wrong sensor information, incorrect interpretation	Robot does not move or take corrective action	Use multimodal inputs, check user intention	Check user attention and intention through multimodal inputs	high
UML 21	R 102	Commission	Robot corrects a valid trajectory or pose	Wrong sensor information, incorrect interpretation	Robot collides with user	Use multimodal inputs, check user intention	Check user attention and intention through multimodal inputs, check pose prior to movement	high
UML 21	R 103	Early	Robot makes trajectory/pose correction during normal operation	Wrong sensor information, incorrect interpretation	Robot collides with user	Use multimodal inputs, check user intention	Check user attention and intention through multimodal inputs	high
UML 21	R 104	Late	Robot corrects a valid trajectory/pose with delay	Wrong sensor information, incorrect interpretation, CPU overload	Robot collides with user, user moves around/impatient	Use multimodal inputs, check user intention	Check user attention and intention through multimodal inputs	high
UML 21	R 105	Value	Robot makes wrong trajectory/pose correction	Wrong sensor information, incorrect interpretation	Robot collides with user	Use multimodal inputs, check user intention	Check user attention and intention through multimodal inputs	high

Table 5: Full hazard analysis results for human error common to any task segment.

Appendix E):

For the hazard analysis of the robot's operation, where the focus is more on what errors the robot can encounter rather than the user, a different approach is taken. Here we include the UML flowchart based on the logical path of actions the robot may take, see Figure 4. At each point or node of the UML (identified with the reference UMLXX) we apply the rigorous technique of the SHARD analysis. This allows creating a correct control architecture and closing the control loop with appropriate sensing suite.

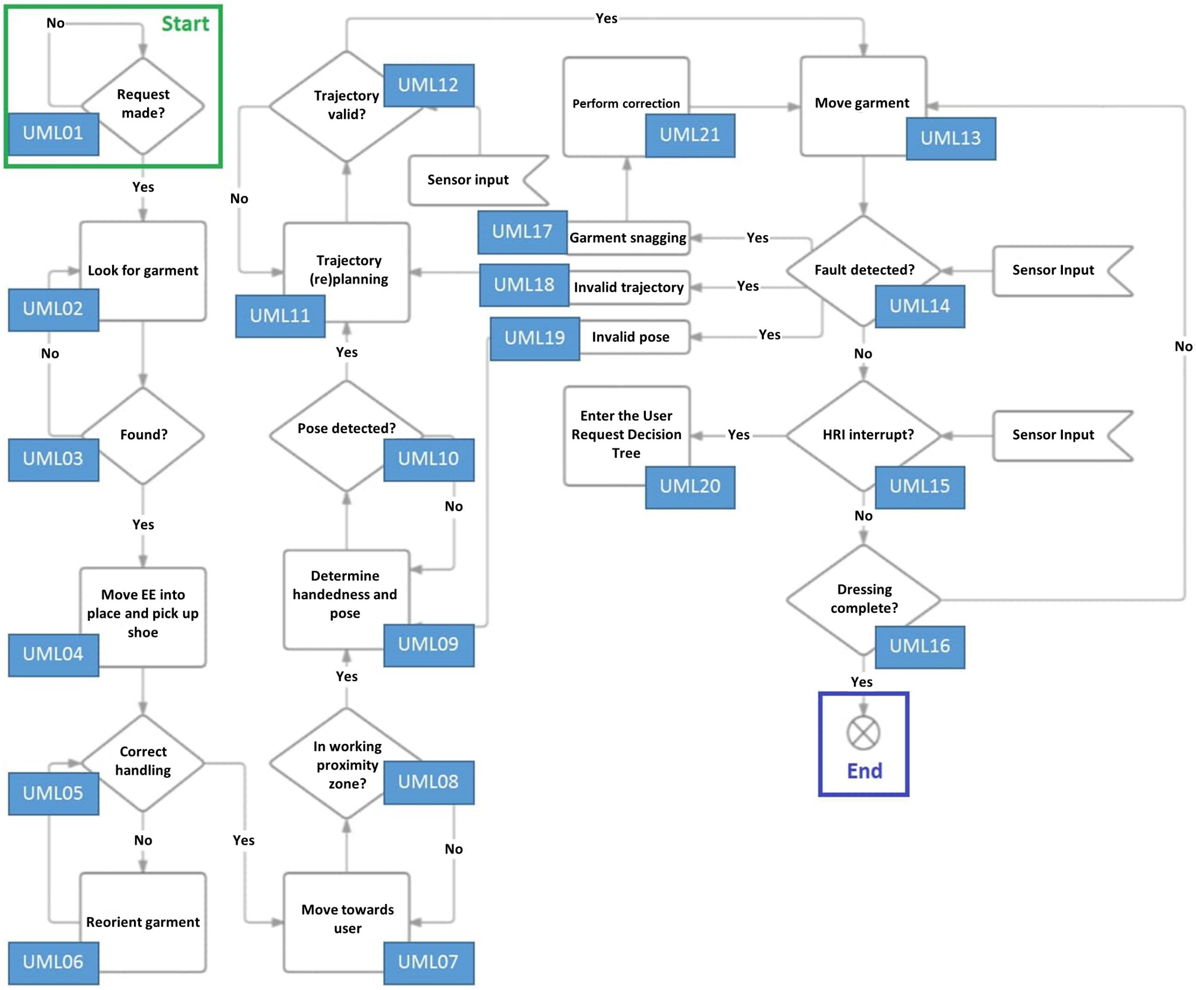

Figure 4: Generic dressing UML diagram with Hazard modules
